# Suicide prevention and ketamine: insights from computational modeling

**DOI:** 10.3389/fpsyt.2023.1214018

**Published:** 2023-06-29

**Authors:** Colleen E. Charlton, Povilas Karvelis, Roger S. McIntyre, Andreea O. Diaconescu

**Affiliations:** ^1^Krembil Center for Neuroinformatics, Center for Addiction and Mental Health (CAMH), Toronto, ON, Canada; ^2^Department of Psychiatry, University of Toronto, Toronto, ON, Canada; ^3^Department of Pharmacology and Toxicology, University of Toronto, Toronto, ON, Canada; ^4^Institute of Medical Sciences, University of Toronto, Toronto, ON, Canada; ^5^Department of Psychology, University of Toronto, Toronto, ON, Canada

**Keywords:** ketamine, computational modeling, psychiatry, generative models, suicidality

## Abstract

Suicide is a pressing public health issue, with over 700,000 individuals dying each year. Ketamine has emerged as a promising treatment for suicidal thoughts and behaviors (STBs), yet the complex mechanisms underlying ketamine’s anti-suicidal effect are not fully understood. Computational psychiatry provides a promising framework for exploring the dynamic interactions underlying suicidality and ketamine’s therapeutic action, offering insight into potential biomarkers, treatment targets, and the underlying mechanisms of both. This paper provides an overview of current computational theories of suicidality and ketamine’s mechanism of action, and discusses various computational modeling approaches that attempt to explain ketamine’s anti-suicidal effect. More specifically, the therapeutic potential of ketamine is explored in the context of the mismatch negativity and the predictive coding framework, by considering neurocircuits involved in learning and decision-making, and investigating altered connectivity strengths and receptor densities targeted by ketamine. Theory-driven computational models offer a promising approach to integrate existing knowledge of suicidality and ketamine, and for the extraction of model-derived mechanistic parameters that can be used to identify patient subgroups and personalized treatment approaches. Future computational studies on ketamine’s mechanism of action should optimize task design and modeling approaches to ensure parameter reliability, and external factors such as set and setting, as well as psychedelic-assisted therapy should be evaluated for their additional therapeutic value.

## 1. Introduction

According to the World Health Organization, suicide is a major public health concern, with more than 700,000 people dying by suicide every year (1), a number that has steadily risen over the past decade (2). Moreover, for each death by suicide, there are more than 20 suicide attempts (1). Individuals suffering from depression are 20-fold more likely to die by suicide compared to the general population (3). In major depressive disorder (MDD), the lifetime prevalence of suicide attempts is estimated to be 31% (4) with completion rates between 5 and 10% (5, 6). However, suicidality is a cross-diagnostic outcome that extends beyond depression alone. Suicidal ideation and attempts can occur in individuals with various psychiatric disorders, as well as in those without a formal psychiatric diagnosis (7). Differentiating between suicidal ideation and suicide attempts is crucial, as the former refers to thoughts and feelings related to suicide, while the latter refers to actual behaviors with potentially lethal consequences. The development of suicidal ideation and the progression from ideation to suicide attempts are distinct phenomena, and researchers have increasingly focused on identifying factors that distinguish between the two (8, 9).

Recent studies have shown that ketamine, an N-methyl-D-aspartate receptor (NMDAR) antagonist, significantly reduces suicidal thoughts and behaviors (STB) (10). Within hours, a single infusion of subanesthetic-dose ketamine relieves depressive symptoms (11, 12), including in treatment-resistant individuals (13, 14), and rapidly and significantly reduces suicidal ideation (10, 15–19). In comparison, treatment with first-line antidepressants, such as selective serotonin reuptake inhibitors (SSRIs), is associated with an improvement in depressive symptoms by the end of the first week, and may take up to 6 weeks to achieve maximum therapeutic effect (20). However, repeated ketamine administrations are required to prolong the drug’s effect, a response which typically abates within a week of a single infusion (21, 22). Notably, ketamine’s anti-suicidal effects may be distinguishable from its antidepressant effects (18, 23, 24). While antidepressant effects are crucial for overall mood improvement, the rapid anti-suicidal action of ketamine can prove critical for those in acute crisis and may hold promise for a broader spectrum of patients with STBs (24). This raises the possibility that ketamine may affect neurobiological pathways associated with STBs independent of depression, emphasizing the unique therapeutic potential of this drug (25).

The replication of ketamine’s rapid antidepressant and anti-suicidal effects has prompted detailed study into the drugs’ pharmacological mechanism of action. Ketamine preferentially acts via NMDAR antagonism giving rise to increased glutamate release from pyramidal cells with subsequent activation of α-amino-3-hydroxy-5-methyl-4-isoxazolepropionic acid receptors (AMPAR). A cascade of downstream signaling pathways is then triggered leading to enhanced brain-derived neurotrophic factor (BDNF) and mammalian target of rapamycin (mTOR) signaling, increased protein synthesis, and synaptogenesis, which may, in turn, promote adaptive rewiring of pathological neurocircuitry (26) [see (27, 28) for detailed reviews of ketamine’s pharmacological profile]. However, ketamine’s effect is not restricted to glutamatergic pyramidal cells, but extends to other neurotransmitter systems, including serotonin, norepinephrine, and dopamine (29–33) as well non-monoaminergic systems (e.g., opioidergic and inflammatory) (14, 34).

Several clinical and neurobiological predictors of ketamine’s antidepressant and anti-suicidal effect have been identified (35–37), however, these data-driven approaches provide little mechanistic insight into how ketamine reduces STBs. This is partly due to the lack of mechanistic understanding of suicidality itself (38). Suicidality is a complex construct that involves multiple factors, such as emotion regulation, cognitive processing, and social and environmental stressors. Advancements in computational psychiatry, namely generative models, hold great potential in unraveling the complexities of suicidality and the mechanisms underlying ketamine’s anti-suicidal effects. Generative models explicitly describe the mechanisms underlying observed neural or behavioral data using biologically-interpretable variables (39, 40). By comparing model simulations against empirical data, the resulting discrepancies can be used to refine the model. This iterative process leads to the development of more informed theories of suicide and compuational models. By leveraging these computational tools, we can gain insight into the dynamic interactions between suicidality and ketamine’s anti-suicidal effects, potentially identifying biomarkers and treatment targets, and enhancing our understanding of the mechanisms involved.

In this paper, we review current computational theories of suicidality and ketamine’s mechanism of action, and discuss various computational modeling approaches being used to understand ketamine-induced changes in STBs. We close with a brief discussion on the implications of computational psychiatry for suicide prevention and address open-ended questions in ketamine therapy research.

## 2. Computational accounts of suicidality

Normative theories of learning and decision-making in computational neuroscience offer a theoretical framework for understanding optimal decision-making processes and can provide insight into the vulnerabilities associated with STB. While computational accounts of STB are only beginning to emerge, there is already a substantial body of literature on cognitive task performance that can inform such accounts.

Several studies have investigated impaired decision-making in individuals with STBs using the Iowa Gambling Task (IGT) and Cambridge Gambling Task (CGT). A meta-analysis of these studies showed an association between history of suicide attempts and riskier decisions compared to both patient controls and healthy controls (41, 42). However, the complexity of the IGT makes it challenging to attribute variations in task performance to specific cognitive processes. Dombrovski et al. (43) addressed this problem by separating choice processes from learning using reinforcement learning (RL) models and a three-armed bandit task. The authors showed that suicide attempters had impaired value comparison and diminished behavioral sensitivity to reinforcement, suggesting impaired reward learning, a deficit that scaled with suicide attempt lethality and was partially explained by poor cognitive control.

In addition to impairments in decision-making processes, decision-making biases may also play a role in STBs (44). Millner et al. (45) used a drift-diffusion model (DDM) and RL model to isolate two decision-making biases in an Avoid/Escape Go/No-Go task, namely active-escape bias (i.e., a bias to “do something” to escape) and an inhibitory-avoid bias (i.e., a bias to “do nothing” to escape). The authors showed that individuals with STBs exhibited a higher bias for active responses to escape an aversive state compared to controls, even after controlling for clinical variables such as depression and hopelessness. However, the inhibitory-avoid bias was comparable between groups, and no significant differences in bias parameters were found between suicide attempters and ideators (45). A recent study by Myers et al. (46), found that reduced inhibitory control, as measured by the Go/No-Go task, was predictive of a suicide attempt within 90 days, and that the miss rate in the same task was a stronger predictor of near-term suicide attempts compared to other commonly used measures. These preliminary findings suggest that decision-making biases assessed through cognitive tasks may hold clinical predictive value.

One overarching explanation of the above findings is an increase in Pavlovian vs. instrumental control (47, 48). Pavlovian control dictates reflexive behavior that rigidly specifies stimulus–response mappings regardless of outcomes. In contrast, instrumental control allows for the adaptation of behaviors to environmental contingencies to achieve desired outcomes in a goal-directed fashion. Increased Pavlovian control can therefore lead to more impulsive actions and biases that are not optimal in the long run. Karvelis and Diaconescu (49) used computational modeling to formally conceptualize the active-escape and Pavlovian biases in suicidality as a product of perturbations in probabilistic learning. According to the model, the Pavlovian active-escape bias and other suicide risk markers, including hopelessness and reduced cognitive control, may stem from the following four mechanistically distinct parameters that capture components of learning and stress responsiveness: increased stress sensitivity, increased learning from stressors, reduced sense of controllability of stressors, and a reduced ability to unlearn maladaptive beliefs. The model was validated by simulating performance in an Avoid/Escape Go/No-Go task, showing that altering each of the four parameters reproduces the findings of increased active-escape bias reported by Millner et al. (45). The authors proposed that these four mechanisms, which represent different hypotheses about the cognitive mechanisms underlying suicidality, may also correspond to distinct subtypes of suicidality. Although the model remains to be tested empirically, the authors’ proposed hypotheses on the connection between suicide neurobiology and cognition/behavior can inform potential mechanisms of action in treatments aimed at reducing STBs (we discuss this in more detail in Section 4.2).

Pavlovian biases may also explain increased loss aversion in STBs. Liu et al. (50) used the balloon analog risk task (BART) to investigate decision-making biases in MDD patients with and without suicide attempts. The study used Bayesian computational modeling to show that the suicide attempter group demonstrated stronger loss aversion than the non-attempter group and healthy controls. The authors posit that increased loss aversion, as a Pavlovian response, may prompt individuals to focus more on current painful experiences and may motivate suicide as a means of avoiding future psychological pain.

Collectively, these findings suggest that impaired value-based decision-making and cognitive control are important factors of suicidal vulnerability (51) [see (48) for a review on impaired decision processes in suicidal behavior], particularly in social and aversive contexts (52). Decision making processes in STBs may be influenced by an affective bias, whereby negative outcomes drive learning more than positive outcomes (53, 54). Suicidal ideation has been associated with a processing bias toward negative stimuli (55), while suicide attempts have been associated with blunted positive affective forecasting for future positive events (56). This may suggest that individuals with STBs fixate on negative outcomes and envision a future with predominantly negative events, and even in the presence of unexpected positive events, their predictions about the future are resistant to change. Over time, an excessive focus on and learning from negative outcomes may lead to a general updating bias toward negative information and result in the formation of overly precise negative prior beliefs (e.g., lower self-esteem, pessimistic worldview). These beliefs can exert excessive influence on one’s thoughts leading to a state of hopelessness—i.e., the belief that no actions can improve one’s situation—and in turn, may result in the adoption of maladaptive action policies, which are defined as strategies used by an agent to determine the next action based on what they have learned. For instance, a reduced sense of behavioral control may foster increased Pavlovian learning, leading to increased Pavlovian biases, where an agent continues to take actions that do not lead to the highest expected reward, such as actively escaping aversive events (45, 49), or avoiding loss (50). This bias is problematic as it can prevent the agent from exploring potentially more rewarding actions and thus hinder the learning of an optimal policy. In a suicidal crisis, vulnerable individuals may respond with increasingly stochastic choices due to impaired reward valuation (43), leading to a misestimation of the value of suicide over superior alternatives (48, 57). Utilizing computational models, one can generate novel hypotheses regarding impairments in learning and decision-making, which can be experimentally tested to develop a mechanistic understanding of the neurocognitive processes that underlie the progression of a suicidal crisis.

## 3. Computational accounts of ketamine

Ketamine has shown promising therapeutic potential in treating suicidal ideation, but its mechanism of action remains unclear. Marguilho et al. (58) recently proposed a unified model of ketamine’s dissociative and psychedelic properties, suggesting that its therapeutic effects are driven by acute modulation of reward circuits and a sub-acute increase in neuroplasticity. More specifically, ketamine may block NMDAR-dependent bursting activity of neurons in the lateral habenula (LHb), a basal ganglia nucleus known as the “anti-reward” center, resulting in the disinhibition of downstream monoaminergic reward centers. Moreover, the sub-acute increase in neuroplasticity, driven by sustained AMPAR activation in excitatory pyramidal neurons and potentiating BDNF and mTOR signaling, allows for sustained antidepressant effects. Additionally, the authors suggest that ketamine’s dissociative and psychedelic properties are driven by dose-and context-dependent disruption of the salience network (SN) and the default-mode network (DMN). The SN, composed of nodes that include the anterior cingulate cortex (ACC) and anterior insular cortex (AIC), helps prioritize relevant stimuli for decision-making and action, while the DMN, composed of nodes such as the posterior cingulate cortex (PCC), medial prefrontal cortex (mPFC), and inferior parietal lobule (IPL), is associated with self-referential thought. Furthermore, functional connectivity between the DMN and ACC has been shown as a key connection for explaining ketamine’s rapid antidepressant action (59). Computationally, the authors propose that nodes of the SN represent high-level priors about the body, and under low doses of ketamine, disintegration of the SN leads to relaxed priors about bodily self-experience thus accounting for ketamine’s ‘dissociative’ effects. At high ketamine doses, disintegration of the DMN leads to relaxed priors about narrative self-experience, accounting for ketamine’s ‘psychedelic’ effects.

The model proposed by Marguilho et al. (58) builds upon earlier predictive processing models describing the mechanisms underlying the therapeutic effects of classic serotonergic psychedelics. The Relaxed Beliefs Under Psychedelics (REBUS) model by Carhart-Harris and Friston (60) and self-binding model of psychedelic ego dissolution of Letheby and Gerrans (61) share the idea that serotonergic psychedelics weaken high-level priors, thereby creating an opportunity for belief revision. The REBUS model integrates theories of the entropic brain and free-energy principle within the framework of predictive coding, and proposes that classic psychedelics induce a heightened entropic brain state under high levels of serotonin 2A (5-HT2A) receptor agonism. This results in increased neural plasticity and a relaxation in the precision weighting of high-level priors or beliefs, possibly through reduced connectivity within the DMN. Belief relaxation, in turn, may enable increased sensitization to bottom-up signaling, rendering aberrant beliefs more amenable to revision (60). Letheby’s self-binding model of psychedelic ego dissolution on the other hand, focuses on ego dissolution by weakening the precision of maladaptive self-representations. The authors identify both the SN and DMN as crucial networks to self-representation, proposing that the SN plays a role with a more minimal or embodied sense of self while the DMN is implicated in higher-level narrative self-representation. Letheby posits that psychedelics reduce the brain’s confidence in expectations about reality and the Self, thereby decreasing their influence on phenomenal awareness (i.e., the subjective experience of the world and oneself), and driving therapeutic change through alterations in self-perception possibly through a reduction in the precision-weighting of high-level priors (61).

All three models converge on the idea that the therapeutic effects of these substances involve the relaxation or weakening of high-level priors and beliefs, which subsequently allows for the revision of entrenched maladaptive beliefs or self-representations (see Table 1 for summary of models). Ketamine may achieve this through a different mechanism than serotonergic psychedelics by directly affecting NMDAR and AMPAR signaling. Previous research has shown that glutamatergic NMDARs facilitate the communication of top-down predictive signals, while AMPARs communicate bottom-up prediction error signals (62–64), and the weighting of these prediction errors is driven by neuromodulators such as dopamine and acetylcholine (65–67). Ketamine’s unique mechanism of action, directly influencing NMDAR and AMPAR signaling, offers a distinct pathway for addressing its therapeutic impact on maladaptive cognitive processes associated with STBs (23).

**Table 1:** Overview of models describing ketamine and/or serotonergic psychedelics mechanism of action.

Model	Key features	Mechanisms
Unified model of ketamine’s dissociative and psychedelic properties (58)	Ketamine’s antidepressant effects are driven by its acute modulation of reward circuits and sub-acute increase in neuroplasticity.	Ketamine blocks LHb bursting activity and modulates ACC circuits leading to disinhibition of downstream monoaminergic reward centers.
	Ketamine induces a dissociative and psychedelic state by relaxing the precision weighting of “bodily” and “narrative” self-representation priors.	Ketamine’s dissociative and psychedelic effects are driven by dose-and context-dependent disruption of the SN and the DMN.
Relaxed beliefs under psychedelics (REBUS) model (60)	Serotonergic psychedelics relax the precision weighting of high-level priors enabling bottom-up information flow and the potential revision of maladaptive priors.	Psychedelics induce a heightened entropic brain state under high levels of serotonin 2A receptor agonism, leading to increased neural plasticity.
		High-level priors may be relaxed through disintegration of the DMN.
Self-model and ego dissolution (61)	Under serotonergic psychedelics, perception of the world and ourselves remains, but attention is allocated differently.	Psychedelic-induced disruption of the SN and DMN are selectively associated with disruption to embodied and narrative aspects of self-representation.
	Experiences are intensified and less personal, and emotional responses, sense of importance, and motivations become detached from personal objectives and history.	

LHb, lateral habenula; ACC, anterior cingulate cortex; SN, salience network; and DMN, default-mode network.

## 4. Computational modeling of ketamine’s anti-suicidal effects

Throughout the remainder of the paper, we attempt to explain ketamine’s anti-suicidal effect using an array of computational modeling approaches. Firstly, we explore its therapeutic potential in the context of the mismatch negativity and predictive coding framework. Secondly, we examine the impact of ketamine on the neurocircuits involved in STBs using Karvelis and Diaconescu’s model of suicidality (49). Finally, we discuss Dynamic Causal Modeling (DCM), a neurobiologically interpretable model that uses neuroimaging data to infer effective connectivity, such as forward and backward connection strengths, among brain regions (68–70). By studying these parameters, we can investigate the altered connectivity strengths and receptor densities implicated in STBs and targeted by pharmacological interventions. The above approaches make use of generative models, which allow for an explicit description of the underlying mechanisms that produce the data. As a result, they provide a detailed disease model and enhance our understanding of the anti-suicidal effects of ketamine.

### 4.1. Ketamine and predictive coding

The auditory mismatch response, or mismatch negativity (MMN), has been widely used to study predictive processing in the brain, and is thought to reflect NMDAR-mediated glutamate function (71–73). The MMN is a measure of pre-attentive sensory processing, and it represents the difference between the brain’s response to a frequently occurring sound (the “standard”) and a less frequent or improbable sound (the “deviant”). The MMN is considered a prediction error signal within the predictive coding framework, which can be used to study the brain’s statistical learning about environmental regularities (74). Essentially, the MMN reflects the brain’s ability to recognize and respond to unexpected or deviant stimuli, which is thought to be important for our ability to adapt to and learn from our surroundings.

The MMN’s potential to serve as a marker for NMDAR function indicates that it may be a useful tool for investigating the role of NMDAR dysfunction in the pathophysiology of depression (75, 76) and STBs (77–79). However, studies of the MMN response in suicidality are lacking, and in depression the results are variable. A recent meta-analysis (80) found that MMN amplitudes to duration deviants, but not frequency deviants, were significantly reduced in depressed patients compared to healthy controls, but depression severity did not correlate with the MMN response. A second review observed a common attenuation in MMN amplitudes of duration deviants and an increase in MMN amplitudes of frequency deviants in depressed patients (81). In contrast, diminished MMN responses have consistently been linked to NMDAR dysfunction in psychosis (82–84), and ketamine has been used to model symptoms of psychosis (85). Several studies have reported reductions in MMN amplitude following ketamine administration (82–84, 86), although the results show substantial variations with ketamine dose, paradigm choice, and trial definition (87–90). Physiologically, a reduction in the MMN amplitude following ketamine administration is thought to be the result of the drug’s inhibition of NMDARs (although concomitant effects of AMPAR function may also play a role).

It is worth noting that the effects of ketamine on STBs using the auditory mismatch negativity response have not been studied extensively. In fact, only one study has investigated ketamine’s antidepressant properties in depressed patients using a frequency auditory roving paradigm. Sumner et al. (91) found increased MMN amplitudes in depressed patients compared to controls 3–4 h post-ketamine infusion, but this effect was only significant when all repetitions of the post-deviant tone were used and was not related to improvements in depressive symptoms (see Section 4.3 for discussion on the study’s DCM results). This finding is in contrast with previous studies that have reported attenuation of the MMN following acute ketamine administration in healthy controls (86). In comparison, Weber, Diaconescu et al. (92) employed a hierarchical Bayesian model of learning to investigate the auditory mismatch response in healthy participants during ketamine infusion, finding that ketamine reduced MMN amplitudes and the expression of high-level precision-weighted prediction errors. These findings suggest that NMDAR inhibition, as an early effect of ketamine, may disrupt high-level inference about environmental volatility. Importantly, it should be emphasized that the timing of the studies was different, with Sumner et al. (91) investigating post-infusion effects during ketamine’s onset of therapeutic action (93) while Weber et al. (92) examined the effects immediately following ketamine infusions. This disparity in the timing could have contributed to the divergent results between the two studies.

Taken together, we hypothesize that ketamine’s effect on the mismatch response may be temporally distinct (75), with reductions in MMN amplitude under ketamine infusion being an immediate consequence of NMDAR blockade, while restored MMN amplitudes may be due to increased sensitivity to bottom-up sensory prediction error signaling via AMPAR upregulation post-infusion. Studies employing ketamine as a model of psychosis commonly perform analyses during the onset of psychotomimetic and dissociative effects, which typically peaks 1-h post-infusion. Hence the ketamine-induced reduction of MMN amplitudes may stem from the drug’s early NMDAR antagonism, while an increased sensitivity to bottom-up prediction error signals via AMPAR up-regulation occurs later during ketamine’s therapeutic time window. To further elucidate the timing effect of ketamine on the MMN response, future studies should consider investigating MMN amplitudes at different time points during ketamine infusion, as well as post-infusion, to better understand the temporal dynamics of NMDAR and AMPAR signaling in relation to ketamine’s anti-suicidal effects.

### 4.2. Neural circuits underlying ketamine’s anti-suicidal effects

The MMN provides valuable insight on perceptual processing and NMDAR dysfunction; however, it does not address important components of STBs, such as stressor controllability and hopelessness. As discussed in Section 2, negative emotional states may lead to negatively biased prior beliefs and a reduced sense of cognitive control. In turn, during times of crisis, stronger Pavlovian influences over goal-directed actions may give rise to the inconsistent valuation of suicide over alternative solutions. According to the suicidality model proposed by Karvelis and Diaconescu (49), a reduced sense of stressor controllability underlying STBs may be associated with the ventromedial PFC (vmPFC) and the serotonin-producing, dorsal raphe nucleus (DRN) pathway. Recruitment of the vmPFC-DRN pathway is thought to promote resistance to stress (94) and the vmPFC has been implicated in value-based decision-making (95). Suicidal behavior and impulsive suicide attempts have been associated with disrupted vmPFC value signals (96, 97). Furthermore, reduced vmPFC connectivity may lead to reduced cognitive control and during a suicidal crisis, this may manifest as a tendency toward stochastic choices such that suicide is chosen at the expense of alternatives (48). Ketamine may enhance stressor controllability by modulating vmPFC activity and increasing DRN serotonin release in the medial PFC (mPFC) (30, 31, 98, 99), which in turn could induce its anti-suicidal effect via activation of the AMPAR/BDNF signaling pathway and a subsequent increase in synaptic function in the mPFC (100).

An additional neurocircuit considered by suicidality model of Karvelis and Diaconescu (49) is the locus coeruleus—norepinephrine (LC-NE) system together with the amygdala, the dorsal PFC, and the anterior cingulate cortex, which may play a central role in mediating learning in response to acute stress and volatility. The importance of NE in the pathophysiology and treatment of depressive disorders is well-established (101), but its specific role in suicide risk remains unclear. Current evidence suggests lower NE function in cases of suicide, characterized by reduced NE transporter binding, decreased density, and a reduced number of NE neurons in the LC of suicide victims (102, 103). Ketamine may promote belief flexibility by increasing NE in the mPFC and modulating the LC-NE system (104–107), potentially facilitating belief updating and making rigid prior beliefs amenable to revision (108). In healthy humans, ketamine has been shown to decrease resting state functional connectivity between the LC and thalamus, potentially increasing nonspecific sensory signal detection (109). Taken together, glutamatergic activation of the LC-NE system by ketamine may promote belief flexibility, allowing individuals to “unlearn” negative prior beliefs and enhance bottom-up sensory information processing.

Karvelis and Diaconescu’s (49) model does not consider dopamine (DA) and there is limited data with variable results on the role of the dopaminergic system in suicidality (110–113). Nonetheless, stressors have significant adverse effects on the mesolimbic DA system, which projects from the ventral tegmental area (VTA) in the midbrain, to the PFC and nucleus accumbens (NAc), among other areas, and functions to regulate reward and salience (114). In animal models of depression, exposure to chronic stress can blunt tonic firing in VTA-DA neurons (29, 115), and according to a recent meta-analysis, ketamine administration restores VTA-DA neuronal activity and increases NAc-DA levels (29). Additionally, ketamine has been shown to regulate VTA-DA activity through upstream modulation of glutamatergic mPFC activity (115), and drive dopamine receptor activation in the mPFC, which may play a role in ketamine’s antidepressant and anti-suicidal effects (116). A recent fMRI study in individuals with remitted depression found that at 2 h post-ketamine infusion during a monetary reward task, brain activity in the VTA during feedback of smaller rewards was positively correlated with levels of (2R,6R)-HNK, an active metabolite of ketamine that directly binds and activates AMPAR (117). Hence, ketamine may improve reward-related deficits via modulation of response to feedback.

Finally, the lateral habenula (LHb) plays a critical role in processing aversive experiences (118) and encoding reward prediction errors (119), and has been implicated in the pathophysiology of STBs (120, 121). As outlined in Marguilho et al. (58) model of ketamine action (see Section 3), ketamine blocks LHb activity (28, 122) and in turn disinhibits downstream monoaminergic reward centers through a relay in GABAergic interneurons in the dopaminergic VTA or the serotonergic DRN. The LHb receives major input from the forebrain, including the mPFC, and may in turn affect processing in the mPFC via VTA and DRN projections (123). Furthermore, the LHb and the ACC may act jointly to mediate decision making and behavioral adjustments during learning, where LHb neurons transmit negative outcome signals to the ACC via dopamine neurons, which accumulates information across trials (59, 123). In the processing of reward and punishment, the LHb may work in tandem with midbrain dopaminergic neurons, signaling prediction errors that arise when reward expectations are violated. Computationally, ketamine may attenuate the salience of unexpected negative feedback and reduce the impact of negative reward prediction errors on belief updates by maintaining tonic firing of the VTA and reducing LHb activity. Greater dopaminergic activity may increase the salience of positive reward prediction errors, promoting greater belief updates from positive stimuli.

**Figure 1:**
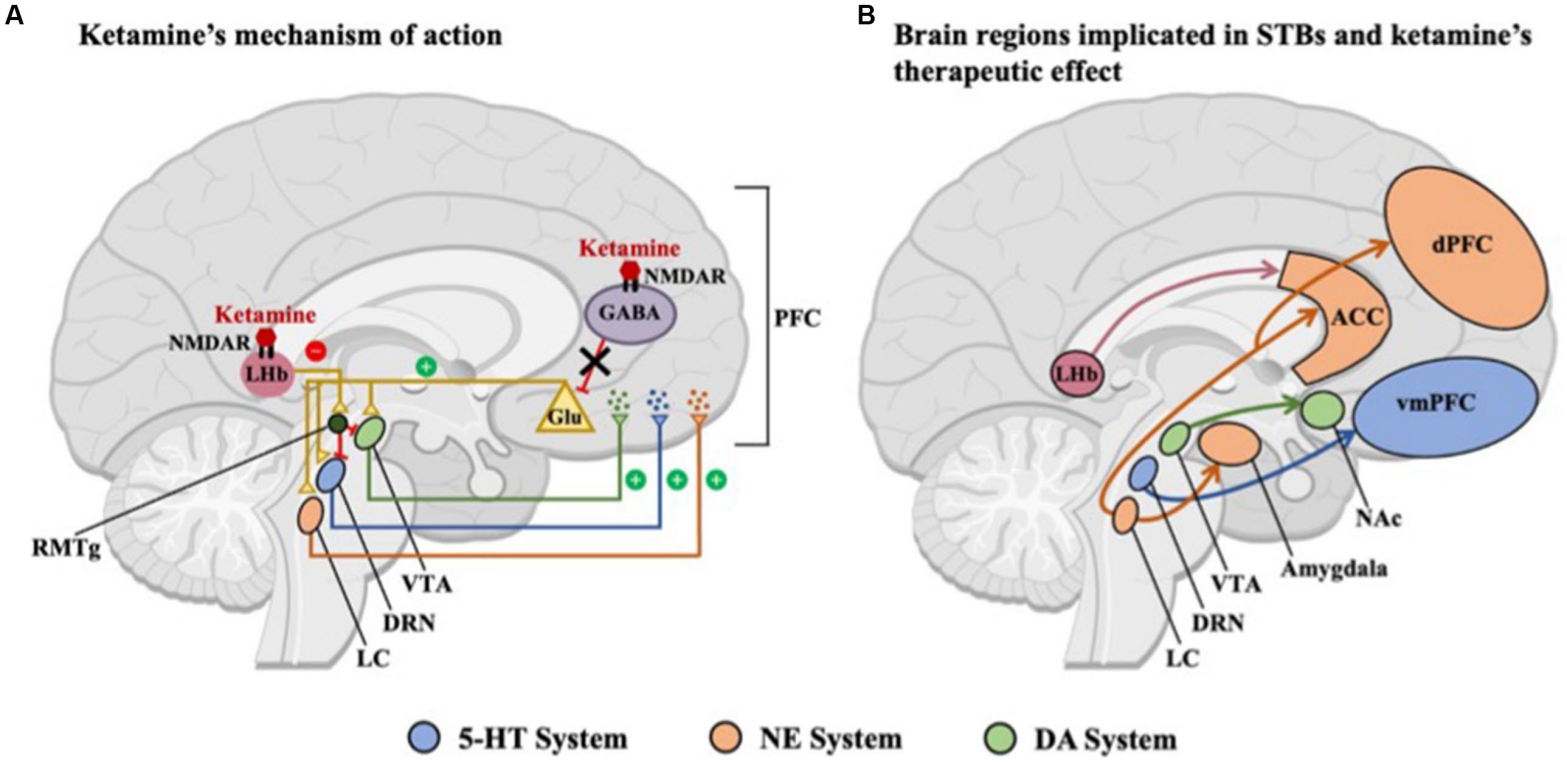
Overview of brain regions included in this paper. **(A)** Ketamine preferentially binds to N-methyl-D-aspartate receptors (NMDARs) located on GABAergic interneurons, predominantly in the medial prefrontal cortex (PFC), resulting in decreased excitability of inhibitory interneurons and as a result, increased glutamate release. Midbrain nuclei, including the serotonergic dorsal raphe nucleus (DRN), noradrenergic locus coeruleus (LC), and dopaminergic ventral tegmental area (VTA), are activated by this glutamatergic surge, leading to the release of monoamines in the PFC. Ketamine also inhibits NMDAR-dependent bursting activity in the lateral habenula (LHb), thus disinhibiting the brain’s reward centers either through a relay of GABAergic neurons in the rostromedial tegmental nucleus (RMTg) or via local interneurons within the VTA and DRN. **(B)** Brain regions and circuits associated with suicidal thoughts and behaviors (STBs), including the LHb and midbrain nuclei, which are indirectly activated by ketamine, including the serotonergic-DRN, noradrenergic-LC, and dopaminergic-VTA. The common activation of midbrain nuclei in both A and B suggests a potential mechanistic link between ketamine’s therapeutic effects and the underlying neural circuitry of STBs. NMDAR, N-methyl-D-aspartate receptor; GABA, γ -aminobutyric acid; Glu, glutumate; NAc, nucleus accumbens; ACC, anterior cingulate cortex; dPFC, dorsal prefrontal cortex; vmPFC, ventromedial prefrontal cortex; 5-HT, serotonin; NE, norepinephrine; and DA, dopamine.

Taken together, ketamine appears to enhance serotonin, norepinephrine, and dopamine signaling, similar to first-line antidepressants [e.g., selective serotonin reuptake inhibitors (SSRI)]. More specifically, ketamine may indirectly increase monoamine release in the PFC through disinhibition of glutamatergic inputs to midbrain nuclei leading to improved feelings of stressor controllability (5-HT-DRN), promote belief flexibility (NE-LC), and improve reward processing (DA-VTA) (Figure 1). The bidirectional activity between the mPFC and these midbrain nuclei may allow for ketamine’s antidepressant properties to persist past its removal from the body (124). Furthermore, increased monoamine signaling has been associated with a strengthening of AMPAR signaling (33, 125) and subsequent BDNF expression (100), all of which is necessary for ketamine’s therapeutic effects. It is important to note, however, that the majority of evidence on the neurocircuitry of suicidality is derived from animal studies and future research should focus on investigating the proposed neural circuits in human populations to confirm and expand our understanding of ketamine’s rapid anti-suicidal effects.

### 4.3. Physiological correlates of ketamine-mediated effects

While computational models of behavior provide valuable insights into learning and decision making, they lack mechanistic physiological explanations of how these processes are implemented in the brain. Conversely, Dynamic Causal Modeling (DCM) describes the dynamics of neuronal populations and aims to explain the directed connectivity changes underlying measured brain activity. DCM has been used to investigate ketamine-mediated effects in healthy controls (88, 126, 127), and only a handful of studies have employed DCM to study the antidepressant mechanisms of ketamine (91, 128–130). Notably, only one study utilized DCM to compare alterations in connectivity estimates associated with suicidal ideation (131).

Sumner et al. (128) conducted an EEG study 3–4 h post-infusion to investigate the effects of ketamine on neural plasticity via visual long-term potentiation (LTP) in depression using DCM. While the study found increased forward and intrinsic connections related to visual LTP, ketamine had widespread effects on forward, backward, and intrinsic connectivity. Notably, both visual LTP and ketamine increased forward connections from the left middle occipital gyrus to the dorsal (left inferior temporal cortex) and ventral (left superior parietal cortex) visual streams. However, due to Bayesian model averaging, the observed connectivity changes could not be directly correlated with the antidepressant response to ketamine. In a separate study by the same group (91), the effects of ketamine on DCM connectivity changes during an auditory MMN task were investigated 3–4 h post-infusion in the same depressed cohort (see Section 4.1 for additional discussion on this study). The study found a significant correlation between an increased forward connectivity from the right primary auditory cortex (A1) to the right inferior temporal cortex (ITC) in response to a deviant tone and a greater antidepressant response 24-h post-infusion. When ITC sources were replaced with superior temporal gyrus (STG) sources in their DCM model, the correlation showed the same trend, but did not reach significance. Both studies found that ketamine modulated forward connections, suggesting an increased flow of bottom-up information, which is consistent with the mechanistic models of ketamine outlined in Section 3.

Schmidt et al. (88) conducted a DCM study on the effects of ketamine on healthy subjects during an auditory mismatch paradigm, and found a significant reduction in the forward connection between left A1 and left STG, with a similar but non-significant trend observed for the homologous forward connection between right A1 and right STG. These results are conflicting with the aforementioned findings of Sumner et al. (91), but as discussed in Section 4.1, the timing of the mismatch task and ketamine’s pharmacodynamic profile may explain this discrepancy. Schmidt et al. conducted their study during an 80-min ketamine infusion, where the reduced forward connection between left A1 and left STG may be an early consequence of NMDAR antagonism. In contrast, Sumner et al. assessed the mismatch response 3–4 h post-infusion, where the increased forward connection between right A1 and ITC may correspond to AMPAR up-regulation.

Multiple studies have utilized DCM in tandem with magnetoencephalography (MEG) to explore the neural mechanisms underlying ketamine’s antidepressant effects 6–9 h post-infusion in treatment-resistant depression (TRD). Specifically, Gilbert et al. conducted three studies in the TRD population to evaluate changes in NMDA-and AMPA-mediated connectivity during somatosensory stimulation (129) and emotional processing tasks (130), as well as resting-state activity (131). Across these studies, an association between AMPAR connectivity and antidepressant response was reported (130, 131), with effects lasting up to 11 days post-ketamine (129). However, these studies used a more liberal criterion of *p* < 0.05 uncorrected to determine significance, which may increase the likelihood of false positives. Nonetheless, these results add support to previous findings demonstrating a key role of AMPAR in ketamine-induced antidepressant effects and underscore the value of DCM for modeling AMPA- and NMDA-connectivity changes associated with ketamine administration and therapeutic response. Additional DCM studies are needed to quantify ketamine’s acute effect in treating STBs, as well as long-term sustained effects.

## 5. Additional considerations and potential applications of computational modeling

While theory-driven computational models offer a promising approach for bridging existing knowledge of suicidality and ketamine’s mechanism of action, multiple questions remain.

### 5.1. Computational models for individual treatment prediction

**Figure 2:**
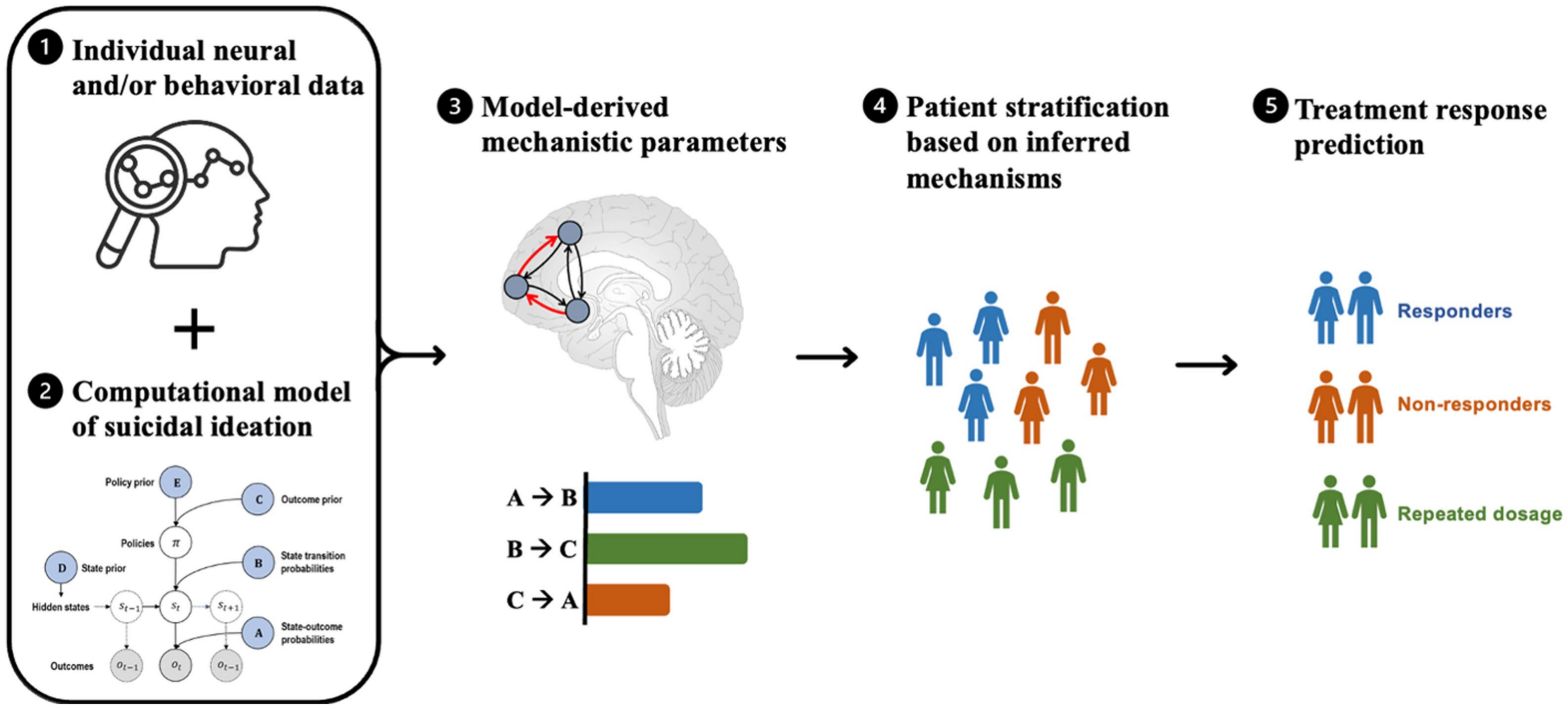
Steps involved in model-based patient stratification and treatment prediction for suicide prevention. The process involves **(1)** collecting brain activity and/or behavioral data from patients, which serves as input to a computational model. **(2)** The computational model is a formally defined model that explains observed neural and/or behavioral data and models various factors relevant to suicidal ideation (e.g., emotion regulation, cognitive processing, and environmental stressors), resulting in a set of mechanistic parameters. **(3)** These mechanistic parameters (e.g., coupling strengths between brain regions, receptor densities, and prediction errors) are used to infer the underlying mechanisms of suicidal ideation in each patient. **(4)** The model-based parameters are then used to stratify patients into subgroups based on similar mechanistic profiles that are most relevant to their symptoms. **(5)** Based on the resulting patient subgroups, the computational model may be used to predict which treatments are likely to be most effective for each subgroup by simulating the effects of different treatments on the underlying mechanisms of suicidal ideation and selecting the treatment that is most likely to produce the desired effects.

Computational modeling enables the assessment of competing mechanistic hypotheses and the generation of biologically interpretable parameters that may be used for model-based patient stratification and treatment prediction (Figure 2). For example, by analyzing behavioral and/or brain data, computational models can be used to link specific mechanisms, such as impaired decision-making and cognitive control, to their underlying neural causes. Subject-specific computational parameters can be used to classify patients into subgroups based on their mechanistic profiles. By simulating the impact of treatments on these mechanistic profiles, the model could predict the most beneficial treatment for each subgroup.

Recently, the combination of computational models of behavior with generative models of neuroimaging data, such as DCM, has allowed researchers to better quantify how prediction errors and learning rates encoded by neuromodulatory systems covary with changes in effective connectivity (132). In turn, mechanistically interpretable features, such as connectivity parameters, can be used as input to machine learning models to predict treatment response – a process known as generative embedding (133). Such models could generate biologically interpretable predictions, and identify patient subgroups who may respond better to targeted medications, such as glutamatergic or dopaminergic treatments.

Furthermore, computational models can be combined with mobile technology to collect real-time data to inform suicide risk and offer a more temporally fine-grained characterization of brain and behavior states (134). Such models may capture sensitive time windows associated with the decline of ketamine’s anti-suicidal effects or the progression of a suicidal crisis and help identify those at imminent risk of suicide. Nevertheless, the use of computational models to generate clinically relevant predictions is still in its infancy, and the reliability and validity of model-derived parameters must be thoroughly tested before they can be used to generate clinically relevant predictions (135).

### 5.2. Computational models for enhancing ketamine’s therapeutic effect

Ketamine’s potential as an antidepressant or anti-suicidal treatment prompts further research into how to optimally harness the drug’s therapeutic effect. For instance, evidence suggests that ketamine’s anti-suicidal effect is not entirely explained by its antidepressant actions (23, 24). Given that suicide is a trans-diagnostic phenomenon, computational modeling could help disentangle the specific mechanisms by which ketamine acts on suicidal patients, both with and without primary mood disorders. Additionally, the absence of a lasting antidepressant or anti-suicidal impact of ketamine, especially in treatment-resistant populations, calls for strategies to extend these effects beyond repeated administration (136, 137). Future research should investigate whether combining psychedelic-assisted therapy with ketamine infusions is necessary for the revision of negative beliefs, the acceptance of emotions, thoughts, and memories, and sustaining therapeutic effects (138, 139). Computational models could be used to quantify long-term changes in cognitive processes associated with this combined therapeutic approach. Lastly, while the intensity of positive “mystical” or “peak” experiences of ketamine have been shown to correlate with greater antidepressant response (140, 141), dissociation may not be necessary for ketamine to be effective (142). Ketamine is often administered at lower doses without the same attention to sets and settings (i.e., preparation, psychological integration, room design, light, and music) that is given to classic psychedelic interventions (143). Differences in administration and preparation procedures may partly explain why classic psychedelics have longer therapeutic effects lasting weeks to months after a single intervention (144) compared to ketamine’s 1 week. Computational models could assist in identifying optimal therapeutic settings and quantifying changes in learning and decision-making associated with ketamine-induced psychedelic experiences (145, 146).

## 6. Conclusion

In conclusion, while the underlying mechanisms of STBs and the anti-suicidal effect of ketamine are still not fully understood, computational models can provide valuable insights into the complex pharmacology of ketamine and its influence on suicidality. However, further studies are needed to optimize modeling approaches and task design, as well as evaluate external factors such as set and setting and the potential therapeutic value of psychedelic-assisted therapy. With continued advancements in computational psychiatry, a more comprehensive understanding of ketamine’s anti-suicidal effects may be achieved, ultimately leading to improved treatment options for individuals at risk of suicide.

## Author contributions

CC, PK, and AD developed the theoretical framework. CC drafted a first version of the manuscript. PK, RM, and AD provided edits and suggestions, and assisted with draft finalization. All authors contributed to the article and approved the submitted version.

## Funding

This work was supported by the Krembil Foundation (to AD).

## Conflict of interest

RM has received research grant support from CIHR/GACD/National Natural Science Foundation of China (NSFC) and the Milken Institute; speaker/consultation fees from Lundbeck, Janssen, Alkermes, Neumora Therapeutics, Boehringer Ingelheim, Sage, Biogen, Mitsubishi Tanabe, Purdue, Pfizer, Otsuka, Takeda, Neurocrine, Sunovion, Bausch Health, Axsome, Novo Nordisk, Kris, Sanofi, Eisai, Intra-Cellular, NewBridge Pharmaceuticals, Viatris, Abbvie, and Atai Life Sciences, and is a CEO of Braxia Scientific Corp.

The remaining authors declare that the research was conducted in the absence of any commercial or financial relationships that could be construed as a potential conflict of interest.

## Publisher’s note

All claims expressed in this article are solely those of the authors and do not necessarily represent those of their affiliated organizations, or those of the publisher, the editors and the reviewers. Any product that may be evaluated in this article, or claim that may be made by its manufacturer, is not guaranteed or endorsed by the publisher.
